# Evaluating the Use of Smart Home Technology by People With Brain Impairment: Protocol for a Single-Case Experimental Design

**DOI:** 10.2196/10451

**Published:** 2018-11-08

**Authors:** Rebecca Jamwal, Libby Callaway, Di Winkler, Louise Farnworth, Robyn Tate

**Affiliations:** 1 Department of Occupational Therapy School of Primary and Allied Health Care Monash University Frankston Australia; 2 Royal Talbot Rehabilitation Centre Austin Health Kew Australia; 3 Summer Foundation Ltd Blackburn Australia; 4 John Walsh Centre for Rehabilitation Research Kolling Institute of Medical Research Sydney Medical School, University of Sydney St Leonards Australia

**Keywords:** assistive technology, clinical research protocol, disabled persons, housing, mobile phone

## Abstract

**Background:**

Smart home technologies are emerging as a useful component of support delivery for people with brain impairment. To promote their successful uptake and sustained use, focus on technology support services, including training, is required.

**Objective:**

The objective of this paper is to present a systematic smart home technology training approach for people with brain impairment. In addition, the paper outlines a multiple-baseline, single-case experimental design methodology to evaluate training effectiveness.

**Methods:**

Adult participants experiencing acquired brain impairment who can provide consent to participate and who live in housing where smart home technology is available will be recruited. Target behaviors will be identified in consultation with each participant based on his or her personal goals for technology use. Target behaviors may include participant knowledge of the number and type of technology functions available, frequency of smart home technology use, and number of function types used. Usage data will be gathered via log-on smart home technology servers. A smart technology digital training package will also be developed and left on a nominated device (smartphone, tablet) with each participant to use during the trial and posttrial, as desired. Measures of the target behavior will be taken throughout the baseline, intervention, and postintervention phases to provide the evidence of impact of the training on the target behaviors and ascertain whether utilization rates are sustained over time. In addition, trial results will be analyzed using structured visual analysis, supplemented with statistical analysis appropriate to single-case methodology.

**Results:**

While ascertaining the effectiveness of this training protocol, study results will offer new insights into technology-related training approaches for people with brain impairment. Preliminary data collection has been commenced at one supported housing site, with further scoping work continuing to recruit participants from additional sites.

**Conclusions:**

Evaluation evidence will assist in planning for the smart technology set-up as well as training and support services necessary to accompany the provision of new devices and systems.

**International Registered Report Identifier (IRRID):**

RR1-10.2196/10451

## Introduction

There is growing recognition that electronic assistive technology (EAT) has the capacity to impact the way support is delivered to people with disability. Recent policy and research highlight that the end user should be empowered and supported to set goals for, choose, and implement the use of EAT, and this person-centered approach will benefit outcomes [1-4]. EAT encompasses mobile computing technologies in use by the wider population as well as specialized devices traditionally designed for and marketed to people with disability [5,6]. Mainstream devices such as smartphones and tablets are emerging as useful tools in developing the independence and participation of people with brain impairment-related disability in a range of life areas [6-9]. Apps that enable home automation and environmental control via these devices continue to emerge [10,11] and present exciting opportunities for this group to exercise environmental control and greater autonomy within the home.

In Australia, new models of supported housing for people with disability are beginning to integrate smart home technologies, including home automation and support staff communication systems, into their base design [12,13]. The use of EAT in these ways is of particular interest to funders of long-term care and support for people with disability, with potential cost benefit associated with reduced dependence on others within the home to complete daily tasks [14]. In Australia, the National Disability Insurance Agency has identified assistive technology, including home automation, environmental control systems, and tablet or smartphone apps, as offering the potential to reduce the lifetime care costs of people with severe disability and reduce the liability to the National Disability Insurance Scheme [2].

Recent research has demonstrated that the opportunities presented by EATs to people with disability living in supported housing have not yet been fully harnessed. A survey of 254 people with disability living in shared supported accommodation (SSA) in Victoria, Australia, demonstrated that only 43.7% had access to mainstream technology, and 10.6% had access to specialized technology [15]. Of 173 multifunction devices in use by surveyed SSA tenants, 42.8% were used for a single purpose and 7.4% had been abandoned (ie, were no longer used by a person) [16]. Qualitatively, interviews with people with acquired brain injury (N=22) living in SSA also indicated that many people were underutilizing the technologies they had access to while others were simply no longer using the devices they had previously purchased or had funded [5]. Participants were consistently more satisfied with the devices they used than they were with the support services they received in relation to the device use [5]. These services included procedures for obtaining the device, repairs or servicing, information and attention when using the device, and continuing support services [17].

Findings across these studies in SSA suggest that the underutilization and abandonment of devices may be attributed to gaps in “soft technology” support services. Soft technologies are defined as the “human factors” that lead to successful device or system uptake, such as any assessment, planning, training, and review involved in locating a suitable assistive technology [18]. Access to 24-hour shared support from disability support workers in SSA was not sufficient to promote and sustain the ongoing technology use for this group. Gaps were identified in support for device selection and set-up in the first instance, as well as ongoing support to modify, develop skills to use, or grade the use of specific devices over time [5]. Similarly, Sohlberg and Turkstra [19] pointed to limited systematic training received as a barrier to the effective use of cognitive aids, which can include EAT devices.

The findings of the above research, when coupled with other investigations, emphasize the need to develop structured approaches for the delivery of technology support services, including a person-centered approach to EAT use and training, for people with brain impairment. Powell et al [20] argued that systematic training delivers better skill maintenance and generalization than trial-and-error approaches. Following task analysis that defines the multiple steps that need to be trained, systematic training involves instructions on how to use the device as well as planning for the support that will promote use in the relevant environment [19]. Ponsford et al [21] similarly emphasized the importance of task analysis prior to multistep training and the importance of delivering interventions in real-life environments, tailored to the needs of an individual. Sohlberg and Turkstra [19] compared the training needs of two people with brain impairment to highlight the differences in training approach required between individuals. The comparison is made between a person with significant memory impairment that impacts his or her ability to learn new information and a person with a brain injury-related executive function impairment that impacts the initiation of device use. The authors suggested that persons with memory impairment may require highly structured, errorless learning approaches that incorporate practice with spaced retrieval. Meanwhile, persons with executive function impairment that impacts initiation may rather require external cues or prompting to use a device in context [19].

The new policy environment of a National Disability Insurance Scheme in Australia has further driven a positive change in consumer empowerment within a market-driven assistive technology environment [2,22]. This further directs the requirement for a person-centered, rather than technology-driven, approach to the uptake and use of EAT. With the need to deliver systematic approaches to technology support for people with brain impairment identified, as well as the opportunity for this group to now move into new models of supported housing with integrated smart home technology, this paper has two aims. First, it will document a systematic training intervention for use with people with brain impairment who have access to smart home technology in their housing. Following this, the paper will outline a multiple-baseline, single-case experimental design methodology that will be used to rigorously evaluate the effectiveness of the training program.

The single-case experimental design offers an intensive research method built on open systematic observation, repeated assessment, and data analysis [23-25]. This study design enables the evaluation of interventions tailored to suit an individual’s needs and is, therefore, a valuable tool in collecting evidence on interventions that can be readily applied to clinical practice [23]. Tate et al’s [26] Model for Assessing Treatment Effect places single-case experimental design at the highest level (level 6) on a hierarchy for evaluating the viability of therapeutic interventions. The following training approach and evaluation protocol aims to positively influence the use of smart home technology, including smart phone- or tablet-controlled home automation or environmental control technology, by people with brain impairment living in technology-enabled housing. The training approach is designed to maximize technology uptake and multifunction use and minimize underutilization or abandonment risk for smart home technology devices and systems.

## Methods

### Research Questions

This project was designed to answer the following research questions:

1. Does smart home technology use increase over time with exposure to this training program, where technology use is defined as both frequency of use of smart home technology and the number of smart home technology functions used?
2. Does awareness or recall of the number and type of features of smart home technology available to an individual resident increase over time with exposure to this training program?

To examine generalization effects, this research will also seek to answer the following research questions:

1. Does exposure to the training program positively influence the psychosocial impact of the smart home technology for the person?
2. Does participants’ satisfaction with their smart home technology increase with exposure to the training program?
3. If positive changes are noted following the training, are these changes sustained beyond the intervention period (to 4 weeks postintervention) and can these changes be attributed to specific phases of the intervention (eg, pre- or postneeds assessment phase, pre- or postsupported practice phase)?
4. Does participation in the training program lead participants to develop further goals for EAT uptake or usage?

The study design and associated participant recruitment as well as data collection procedures have been approved by Monash University’s human research ethics committee.

### Participants

Participants will be people aged >18 years with acquired brain impairment and associated memory impairment who reside in supported housing that offers integrated smart home technology and 24/7 staffing support available on-call, either onsite or remotely. Residents of these supported housing models who use the integrated technology will be invited to participate in this study. The key inclusion criteria of this study are that participants have identified goal(s) to increase their technology use. People identified through the initial “permission to contact” stage of a third-party recruitment strategy (see below) who do not wish to increase their technology use will not be recruited into the study.

In this study, a third-party recruitment strategy will be used. An invitation to participate (and contact details “permission to contact” slip) will be provided by the research group to a representative of the disability support provider at the housing model. This provider will pass the invitation on to eligible residents so they can consider whether they would like to release their contact details to the research group. This study will only include participants who can provide their own informed consent. The service provider handing on the invitation will know whether the person can provide his or her own consent to participate, as part of their service agreement with the resident. It is then the individual resident’s decision as to whether he or she would like to fill out the permission slip and post or email a copy of it back to the research team so that the research group can contact him or her to provide more information about the study. This recruitment design ensures that the research group only accesses a person’s contact details if the individual chooses to release them and that the service provider need not know who has, or has not, responded.

### Intervention Setting

This training intervention will be carried out in the homes of consenting participants.

### Target Behavior

The following are the target behaviors of this intervention:

1. Frequency of smart home technology use each day
2. Number of smart home technology function types used, which may include the following functions: lights on or off, blinds up or down, door open or close, heater or cooler on or off
3. Participants’ knowledge of the number and type of smart home technology functions they can access

Data on frequency and number of functions in use will be gathered daily via actual logged data stored on the smart home technology server at the chosen housing site. Measures will be taken throughout the baseline, intervention, and postintervention phases to provide evidence of the impact of the training on the target behaviors and to ascertain whether utilization rates of the integrated technology are sustained over time. In addition, emphasis will be placed on developing an integrated digital training package that can be left with the persons with disability (or where appropriate, a family member or other carer) so that they can be referred back to if required in future.

### Baseline Measurement

The frequency of use and the type of smart home technology function used is logged by the server controlling the smart home system every time a function is used (including the time and date it is used). Data can be extracted from the server with the consent of the resident via a system-generated log report provided by the technology company that installed the system. In addition, baseline data on participants’ frequency and type of technology use will be gathered daily over 7 days via review of these system-generated logs, thus, providing 7 baseline data points. In this way, the research team will be provided with a thorough understanding of participants’ preintervention frequency and type of technology use. Furthermore, a review of data logs will take place prior to each intervention session to monitor participants’ progress.

A short questionnaire will be used to gather baseline data on the participants’ knowledge of the number and type of smart home technology functions they can access at their home. The questionnaire will commence using a free recall format, asking participants to name each function that can be controlled using the installed smart home technology (eg, “Can you please tell me all of the functions that you are able to control using your smart home technology?”). Following this, participants will be presented with a list of functions that may be controlled by the smart home technology; this list will also contain distractor items or functions that are not controlled by the smart home technology installed in participants’ home. In addition, participants will be asked to select the functions that their smart home technology can control (eg, “What functions are available on your device? Please tick all that apply”). Data on the number of functions named correctly or incorrectly via free recall will be collected along with data on the number of functions selected correctly or incorrectly via the prepared list. Furthermore, the questionnaire will be customized to the housing site within which the research takes place to ensure that all smart home technology functions available are captured and appropriate distractor items are included.

Once these baseline data have been collected, participants will be oriented to the range of functions that their smart home technology can be used for via a prepared list that participants will keep. Participants will be supported to post this list on a prominent place (eg, a noticeboard or fridge) to refer to between sessions. Use of this short questionnaire will continue throughout the intervention to explore whether participants’ knowledge and awareness of the number of functions available increases from baseline.

In this study, we will use a concurrent multiple-baseline design across 3 participants. According to Kazdin [25], typically ≥3 baselines are used in multiple-baseline design research to demonstrate the impact of the intervention applied. As per this method, baseline data will be collected continuously and concurrently for 3 participants, and the intervention will be introduced in a staggered or time-lagged fashion across participants at different time-points. Specifically, the training package will be implemented with the first participant while baseline data gathering continues with the remaining participants. The training package will be implemented with a second participant once the first participant shows that the intervention is impacting his or her technology use. This process will be repeated before a third participant is added to the intervention phase. As described by Kazdin [25], staggering the start of an intervention is undertaken to ensure that it is the intervention that is responsible for the change, rather than other external factors. The design effectively minimizes threats to internal validity, such as history and maturation.

### Equipment

#### Smart Home Technology

Participants may access the smart home technology features integrated within the design of their supported housing via a number of smart technologies that include iOS smartphone, Android smartphone, and iOS or Android tablet.

These devices may be used by participants for other reasons beyond smart home technology functionality (eg, electronic social networking). Each device is smart home technology-enabled via a software app, and participants may use multiple devices to access this functionality (eg, a combination of smartphone and tablet). This app is loaded on to the smartphone or tablet to be used and provides a software-user interface for residents to access and control their smart home technology. A brief software operating guide, detailed operating guide, and step-by-step flowchart guide will be developed by the research team, specific to the software app in use at the housing site. Note that in the absence of smart devices, the smart home technology features can be operated by residents or support staff manually via wall-mounted switches or standard remote controls (eg, air conditioner remote control).

#### Video Training Tool

A video training tool will be developed in consultation with each research participant; it will target priorities for smart home technology use identified by the individual participant. The video training tool will consist of text, audio, and video components, providing a step-by-step demonstration to participants for using each prioritized technology function. Furthermore, video footage will be captured by the research team and edited using a Web-based video production tool to include step-by-step audio and text. This training tool will be saved to the smartphone or tablet that the person uses so that he or she can refer to it between sessions, show it to informal or paid support persons, or continue to view or share it with others once the intervention is complete, if desired.

### Intervention Phases

**Figure 1:**
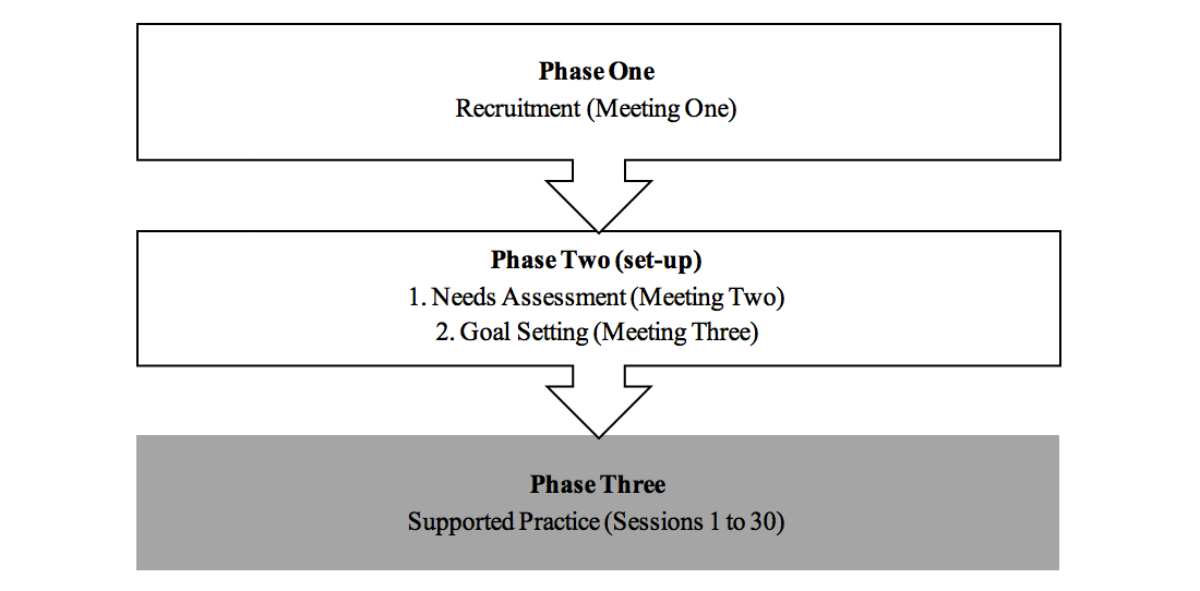
Intervention phases; gray cell denotes the active intervention phase.

Figure 1 shows the 3 intervention phases of the study.

#### Phase One

##### Meeting One—Recruitment

We will follow project explanation and informed consent process using human research ethics committee-approved forms. Once consent is provided, the World Health Organization Disability Assessment Schedule 2.0 [27] will be administered to document the functional status of participants. Then, baseline data recording will commence. Data logs will be downloaded from the server via the technology provider for 7 days.

#### Phase Two

##### Meeting Two—Needs Assessment

Meeting two will be conducted with one participant at a time and only once baseline usage data have been collected.

1. Two published measures examining the experience of technology use will be used to collect preintervention data: the Psychosocial Impact of Assistive Devices Scale (Question 3) [28] and the Quebec User Evaluation of Satisfaction with assistive Technology (Question 4) [17].
2. A short questionnaire to ascertain participants’ preintervention understanding of the functions available on their assistive technology will be administered. Specifically, this questionnaire will ask participants to use free recall to name all of the functions available and, then, ask participants to select all of the functions available from a prepared list that contains distractor items.
3. Participants will be oriented to the number of functions available on their smart home technology, verbally and with a numbered list, which will be left with the person on a prominent place that they can view between sessions. Any barriers to technology use will be identified and ameliorated if possible.

If there is a major barrier to participants’ technology use, for example, ineffective mounting of the device to participants’ motorized wheelchair, an occupational therapist will assess and recommend a solution prior to the start of the intervention.

##### Meeting Three—Goal Setting

The events below will take place during meeting three. Goal setting will be undertaken, which will involve the following:

1. Reviewing the number of functions available on the device with participants—verbally and with a numbered list
2. Comparing the baseline data that show the frequency and type of current smart home technology function use against all the functions available on the smart home technology system to determine whether there are any functions that are not in use, or that are utilized infrequently, that participants may be interested in using. Alternative control methods in use (ie, wall-mounted switch or standard remote controls) will be discussed with participants, and the preferred method of control will be confirmed.
3. If more than one function is identified, the functions that the person would like to increase their use of via their participation in this intervention program will be prioritized (to a maximum of 3 functions). A visual reminder of the prioritized functions will be prepared and left with participants on a prominent location for them to review between sessions. This visual reminder of prioritized functions will be incorporated within the list of all 12 functions previously prepared.
4. Preparation of video training tools to act as a step-by-step guide to using the prioritized technology function(s)
5. Preparation of a low-technology (paper-based) flowchart that will be used to prompt participants on the steps to navigate to the function(s) if they are unsuccessful in navigating the user interface to locate and activate the nominated smart home technology function(s) after viewing the video twice

#### Phase 3—Supported Practice

According to Sohlberg and Mateer [29], “consistent practice and support for evaluating one’s performance can result in improved error recognition and correction” [pg 255] following acquired brain injury. Short, frequent sessions will, therefore, be utilized within this phase to provide participants with multiple opportunities to practice their technology use in the presence of a disability support worker who can provide feedback. A trained disability support worker having experience working with people with brain injury and related cognitive impairment will be employed to deliver these practice sessions. Sessions length will range between 20 and 30 minutes every second day (including one weekend day).

The duration of the training program will be guided by participants’ achievement of the mastery criterion, that is, when a participant demonstrates an increased usage of the smart home technology functions (target behaviors) he or she prioritized for the intervention. The duration of the training program will not extend past 10 weeks.

### Treatment Adherence

Training sessions will be video- and audiotaped to allow an assessor (an independent member of the project team) to rate whether the disability support worker administered all components of the training program as outlined in the intervention protocol.

### Session Guide

#### Session One

At the beginning of the first intervention session, participants will be asked, in free recall format, to name all the functions that can be controlled by the smart home technology installed in their home. Participants will then be provided with a list containing each of the smart home technology functions available, as well as distractor items. Next, participants will be asked to select the items that their smart home technology can control. Responses will be recorded and counted to compare against the number of functions available and the number of functions identified by participants in Meeting Two at baseline. In addition, participants will be provided with feedback on their responses to this question and, if required, reoriented to the number of functions their technology can be used for. These data will be compared with data gathered during the intervention period, providing evidence as to whether the intervention has expanded participants’ awareness or recall of the number of smart home technology functions available to them. Furthermore, these data will be compared against actual technology use to determine whether there are any differences between the knowledge of what technology can be used for and the actual use.

The disability support worker will then ask participants to identify which function it was that they had prioritized to focus on within the training intervention. The disability support worker will prompt participants to check the visual reminder developed in the previous session if they are unable to recall this. The disability support worker will demonstrate how to access the link to the video training tool, saved in a “memo” on the person’s smart device. Participants will then be prompted by the disability support worker to view the video of the first smart home technology function they prioritized in the goal setting interview. They will then practice using it once while the disability support worker is present. The disability support worker will make a record of the steps completed successfully using a progress monitoring form (Multimedia Appendix 1), providing immediate feedback to participants on their performance once they have completed their first attempt. If a participant makes an error in navigating through the user interface, locating the desired function on the device or activating the function, and does not initiate self-correction, the disability support worker will immediately provide feedback and prompt the participant to review the video for a second time. If the participant is unable to navigate the user interface, locate, and activate the desired smart home technology function after viewing the video for a second time, the disability support worker will provide the person with the paper-based step-by-step guide for navigating, locating, and activating the desired function and use this in conjunction with verbal prompting to guide the participant through each step of using the nominated smart home technology function.

Instances in which a participant requires subsequent review of the video or the paper-based written and verbal prompting will be noted on the progress monitoring form. This procedure of prompting incorporates components of errorless learning, modeling the skill before participants attempt it (via video tool), and providing immediate feedback if participants make an error. These strategies have been included in this study to ensure a high rate of accuracy during the acquisition phase of learning. Given the goal of the acquisition phase of learning is to improve the accuracy of skill performance, this training program has been developed to support participants to experience correct use of the function and to avoid participants internalizing memory of incorrect use [29]. The flowchart will be designed so that it can be kept on the inside cover of the iPad, or at another accessible place, depending on the needs of participants.

#### Subsequent Intervention Sessions

At the beginning of subsequent intervention sessions, participants will be asked, in free recall format, to name the functions that they can control using their smart home technology. Then, participants will be provided with the list used in intervention session one and asked to select the functions that their smart home technology can control. Data will be recorded and counted to compare against the number of functions available and the number of functions identified by participants at baseline. Participants will be provided with feedback on their responses to this question and, if required, reoriented to the number of functions the technology can operate (using the existing list).

The reflection-prediction technique, a metacognitive strategy, will be utilized within each remaining session to allow participants to compare their predictions of performance with actual performance. According to Sohlberg and Mateer [29], “this process gives the client information about his or her real-world functioning in such a way that the client can learn more about what is working and not working” [pg 289]. Use of the reflection-prediction technique in this study will involve the review of performance since the previous session, at the start of the next session that immediately follows. This process will assist to identify behaviors that have been targeted successfully and highlight areas that require further practice. The disability support worker will utilize the following script to facilitate the reflection-prediction technique:

- Do you think your technology use has changed since we last met?
- I have some data here on your technology use since we last met. It looks like the frequency of your technology use has (increased or decreased or stayed the same), and it looks like you are using (more or less or the same) types of functions within your smart home technology. Since our last session you are using X, Y, and Z. Why do you think your technology use has changed or stayed the same or decreased since the last session?
- Have you been using the video outside of the intervention? The video has had (x) views. Were any or all of those views by you?

Cumulative review, defined as a regular review of previously learned skills [30], will then be used to review the content covered in the last session using probes including the following:

- What function did you focus on in the last session?
- Can you show me the steps to using [the function focused on last session]?

The disability support worker will prompt participants to review the part of the video training tool that demonstrates this function if they are unable to demonstrate the correct use of the function during this review phase of the session.

If participants have increased the frequency of use of their first prioritized technology function and can successfully demonstrate its use in the review phase, they will be prompted by the disability support worker to address the second prioritized function. As in session one, the disability support worker will provide feedback within the session on participants’ performance, aligned with the principles of errorless learning. If a participant makes an error, the disability support worker will prompt the participant to review the video for a second time. If the participant is unable to use the smart home technology function after viewing the video for a second time, the disability support worker will provide the resident with the printed step-by-step flowchart to assist with navigating, locating, and activating the desired function and use this in conjunction with verbal prompting to guide the participant through each step of using the smart home technology function. Steps completed successfully and instances in which a participant requires a second review of the video, or written and verbal prompting, will be noted on the progress monitoring form.

The procedure explained above will be repeated session after session until each of the participant’s technology use priorities have been addressed.

#### Postintervention Follow-Up (4 Weeks Postintervention)

Four weeks following the cessation of the intervention, system-generated data logs will be reviewed to check whether the person has maintained the frequency and type of technology use that was logged at the end of the intervention. A final meeting will be arranged with participants to discuss the data log and their perspective on the effectiveness of the training intervention and any changes to their technology use over that time since the end of the intervention, including whether participants wish to further increase their use of the smart home technology system or explore other EAT device uptake options. Questions regarding whether the training tool has been used since the intervention and, if so, by whom and how often will also be asked. Furthermore, video “view” data will be recorded. The Psychosocial Impact of Assistive Devices Scale and Quebec User Evaluation of Satisfaction with assistive Technology will be administered for the final time to detect any changes in psychosocial impact and satisfaction since the cessation of the intervention.

## Results

This project has received funding from the Transport Accident Commission, through the Institute for Safety, Compensation and Recovery Research. The Institute for Safety, Compensation and Recovery Research is a joint initiative of the Transport Accident Commission, WorkSafe Victoria, and Monash University. An initial 2 participants underwent written explanatory and consent processes and provided signed consent to participate in the study. These participants were from a single apartment development that offers tenants access to home automation and communication technologies. After the collection of demographic data, initial testing of the home automation technology server indicated that data were not being reliably recorded. The technology supplier was contacted to rectify this issue. The consenting participants were advised that data collection would be placed on hold. Further participant recruitment and data collection will be resumed when this server issue is rectified at the specified site. In the meantime, scoping work for recruitment of additional participants at alternate sites offering the necessary server data collection is being undertaken.

## Discussion

The availability of smart home technology, and recognition of the utility of such technologies for people with disability, continues to grow. There is a need to ensure that people with access to such technology are able to maximize device usage, as desired, so that the opportunities presented by these devices and systems are not lost. The above training intervention was designed to be implemented collaboratively with people with brain impairment who wish to maximize the use of the smart home technology made available to them in supported housing and, thus, ensure that the underutilization and abandonment of these technologies can be avoided. The design of this single-case experiment protocol meets the requirements of level 6 on the Model for Assessing Treatment Effect hierarchy [26]. Target behavior measurements are to be taken frequently and repeatedly in both the baseline and intervention phases, and treatment delivery can be staggered using a multiple-baseline approach. In this way, any cause-effect relationships between the intervention and target behaviors can be demonstrated [31]. The existing literature in the area of smart home technology includes descriptions of the types of technology available [6,7] and research that has evaluated residents’ experience of integrated technology in housing [12]. However, the existing literature does not report on specific interventions that may support smart home technology uptake and use. To the best of the authors’ knowledge, this is the first systematic training program targeting smart home technology uptake and use by people with brain impairment to have been documented, which also presents a single-case experimental design methodology for its evaluation.

The training activities proposed for use in this trial were designed to be as clinically accessible as possible while still grounded in learning theory derived from practice and research on people with acquired brain impairment [19-21,29]. A Web-based video production tool was selected as an easily accessible and low-cost platform that would enable the packaging of audio, video, and text into a single training resource that can then be viewed and shared by the end user—residents using smart home technology in housing. Furthermore, the use of such a platform means that individualized, video-based training resources can be developed to meet the unique needs of each participant in a cost-effective manner. All other resources to be developed as part of the intervention are low tech in nature (eg, paper-based flowchart) and can be easily adapted to the needs and smart home technology goals of each participant. The theoretical background of this training approach, incorporating the use of evidence-based metacognitive strategies and errorless learning principles [19,29,30], supports its potential effectiveness. Data will be collected on participants’ actual usage, their awareness of functionality availability, their satisfaction with the smart home technology, and psychosocial impact of use, providing a wide-ranging examination of the impact of this training program.

This single-case experimental design protocol clearly defines the behaviors to be targeted via the training intervention and the ways in which these behaviors will be repeatedly measured. According to Tate et al [32], the reliability of the target behavior measures used is an important consideration. The opportunity to use a computer server to objectively log or record data on participants’ smart home technology use may overcome potential challenges presented by the use of human raters, who may not be able to record actual use data as accurately. The analysis of this “big data,” automatically recorded by many smart home devices and systems [33], enables researchers to quantify actual use and monitor any changes that occur as a result of the intervention. While conducting ethical human research, it is paramount that participant consent is obtained prior to the collection and analysis of this private data. Furthermore, so that the reliability of this data can be assured, data recorded via the computer servers at housing sites will need to be reviewed for accuracy, prior to participant recruitment and intervention commencement in this study.

Findings garnered from the future trial of this training approach will present important considerations for therapists who prescribe EAT to people with brain impairment. The findings of this research should guide therapists in planning for technology support services that must accompany the provision of any assistive device to clients, including smart home technology. In doing so, therapists can contribute to the longevity and utility of assistive technology solutions for their clients. Furthermore, documenting this intensive training approach offers the potential to provide the evidence of EAT usage and capacity building to the funders of EAT, including state-based insurers and the National Disability Insurance Agency. This evidence includes the amount and type of support services required to ensure the effective uptake and sustained use of EATs beyond the investment that needs to be made in the device or system itself.

## Appendix

Multimedia Appendix 1 Progress monitoring sheet.

## Abbreviations

EAT: electronic assistive technology
SSA: shared supported accommodation
