# Supplementary material for: Evaluating the Use of Smart Home Technology by People With Brain Impairment: Protocol for a Single-Case Experimental Design
Source: JMIR Res Protoc. 2018 Nov 8;7(11):e10451. doi: 10.2196/10451 (PMC6258092; doi:10.2196/10451)
Supplement: Multimedia Appendix 1 [file resprot_v7i11e10451_app1.pdf]

Appendix A: Example of Progress Monitoring Form

Task: Turn (insert function) on

		Session Data				
Steps	Session Date (dd/mm/yy)					
Press device home button	First attempt ✓/✕					
	With second video review ✓/✕					
	With step by step guide ✓/✕					
Swipe to unlock device	First attempt ✓/✕					
	With second video review ✓/✕					
	With step by step guide ✓/✕					
Locate home automation app icon	First attempt ✓/✕					
	With second video review ✓/✕					
	With step by step guide ✓/✕					
Tap app icon	First attempt ✓/✕					
	With second video review ✓/✕					
	With step by step guide ✓/✕					
Tap "CONTROL"	First attempt ✓/✕					
	With second video review ✓/✕					
	With step by step guide ✓/✕					

Tap (insert function)	First attempt<br>✓/✕					
	With second video review<br>✓/✕					
	With step by step guide<br>✓/✕					
Tap “ON”	First attempt<br>✓/✕					
	With second video review<br>✓/✕					
	With step by step guide<br>✓/✕					
